# Wet-Chemical Preparation of TiO_2_-Based Composites with Different Morphologies and Photocatalytic Properties

**DOI:** 10.3390/nano7100310

**Published:** 2017-10-09

**Authors:** Liqin Xiang, Xiaopeng Zhao

**Affiliations:** Smart Materials Laboratory, Department of Applied Physics, Northwestern Polytechnical University, Xi’an 710129, China; lqxiang@nwpu.edu.cn

**Keywords:** TiO_2_-based materials, photocatalysis, nanomorphology, preparation

## Abstract

TiO_2_-based composites have been paid significant attention in the photocatalysis field. The size, crystallinity and nanomorphology of TiO_2_ materials have an important effect on the photocatalytic efficiency. The synthesis and photocatalytic activity of TiO_2_-based materials have been widely investigated in past decades. Based on our group’s research works on TiO_2_ materials, this review introduces several methods for the fabrication of TiO_2_, rare-earth-doped TiO_2_ and noble-metal-decorated TiO_2_ particles with different morphologies. We focused on the preparation and the formation mechanism of TiO_2_-based materials with unique structures including spheres, hollow spheres, porous spheres, hollow porous spheres and urchin-like spheres. The photocatalytical activity of urchin-like TiO_2_, noble metal nanoparticle-decorated 3D (three-dimensional) urchin-like TiO_2_ and bimetallic core/shell nanoparticle-decorated urchin-like hierarchical TiO_2_ are briefly discussed.

## 1. Introduction

Based on its unique chemical and physical characteristics, titanium dioxide (TiO_2_) has attracted much attention in many fields including paint pigments, photocatalysis, solar cells, antibacterial agents, electrical energy storage and some advanced functional materials [1,2,3]. The performance in these applications strongly depends on the microstructure, crystallinity and nanomorphology of TiO_2_ [1]. In particular, in the photocatalysis field, although a new type of polymeric photocatalyst—that is, graphitic carbon nitride—has been intensively investigated recently due to its huge advantages—including metal-free contents, visible light absorption ability, suitable band gap for water splitting, stability, and being environmentally benign [4,5,6,7]—TiO_2_ is still regarded as one of most ideal candidates for photocatalysis because of its strong oxidization, harmlessness to surroundings, chemical inactivity, good stability and low cost [1,3,8,9]. There are three important processes including photo excitation, bulk diffusion and the surface transfer of photoinduced charge carriers in photocatalysis [8]. Thus, the performance of photocatalysis depends strongly upon the charge transfer at the material surface and the light-response range of materials [1,10,11]. The processes of light harvesting and charge transfer efficiencies are affected mainly by the size, crystallinity and nanomorphology of TiO_2_ materials [1,8,10,11,12,13,14]. The preparation and photocatalytic properties of TiO_2_ with different morphologies including zero-dimensional (micro/nanospheres), one-dimensional (rods, tubes, and nanowires), two-dimensional (films, layers and sheets), and three-dimensional (porous spheres, urchin-like spheres) TiO_2_ structures have been widely investigated in the past decade [15,16,17,18,19,20,21]. Different ways have been developed for preparing TiO_2_ materials with different nanostructures. The general synthesis approaches for the fabrication of TiO_2_ materials include sol-gel, hydrothermal and solvothermal techniques [1]. Controlling the microscopic structures of TiO_2_ is still a challenge because TiO_2_ precursors are highly reactive.

Pure TiO_2_ is not a perfect photocatalyst due to the disadvantages of low photocatalytic efficiency and the narrow light-response region [8]. Doping metal ions or introducing noble metal nanoparticles onto the surface of TiO_2_ was demonstrated to be one of the effective ways to enhance the photocatalytic efficiency because these TiO_2_-based composites can combine the functions of TiO_2_ and metal ions or noble metals [22,23,24,25]. Furthermore, the properties of the composites can be adjusted by controlling the ingredients and the microstructures of the TiO_2_.

In the past two decades, our group has focused on the synthesis, electrorheological (ER) properties, luminescence properties and photocatalytic activities of TiO_2_-based materials [26,27,28,29,30,31,32]. A series of TiO_2_-based materials with different compositions, crystallinity and interior microstructures have been synthesized by different methods [33,34,35,36]. The TiO_2_ particles with a familiar microstructure, such as solid spheres [37], hollow spheres [29], porous spheres [28,38], hollow porous spheres [39] and urchin-like spheres [30], were synthesized and characterized in detail. In addition, some TiO_2_ composites with a special interior microstructure were also designed and synthesized [40,41,42,43]. According to the dielectric design, rare-earth-doped TiO_2_ particles were synthesized by sol-gel methods [44,45,46,47,48,49]. Inspired by the structure of biological surfaces, a kind of composite particle possessing both nano- and micro-scale structures was prepared via a hydrothermal method [50,51]. TiO_2_ particles with a cell-like structure were also synthesized [52]. It is noteworthy that the TiO_2_-based materials described above show excellent properties in different applications. For example, the rare-earth-doped TiO_2_, and the micro- or nano-structured composites with TiO_2_ have been demonstrated to show a distinct enhancement in their ER properties [44,50,51,52]. The hollow Sm^3+^-doped TiO_2_ and the monodisperse mesoporous Eu-doped TiO_2_ spheres have shown good luminescent performance [28,29]. The urchin-like TiO_2_ and urchin-like TiO_2_ decorated with Au, Ag, Co@Au or Co@Ag nanoparticles have shown significant improvement in photocatalytic activities [30,31,32].

Until now, there have been many review articles introducing the progress made in the field of TiO_2_-based materials [1,2,3,8]. Based on our group’s research work on TiO_2_ materials, this review is primarily concentrated on the preparation of TiO_2_ composites with different morphologies and the photocatalytic activities of urchin-like TiO_2_ composites.

## 2. Preparation of TiO_2_ and TiO_2_-Based Composites with Different Morphologies

### 2.1. Micro- and Nano-Spheres

#### 2.1.1. Solid Spheres

**Figure 1:**
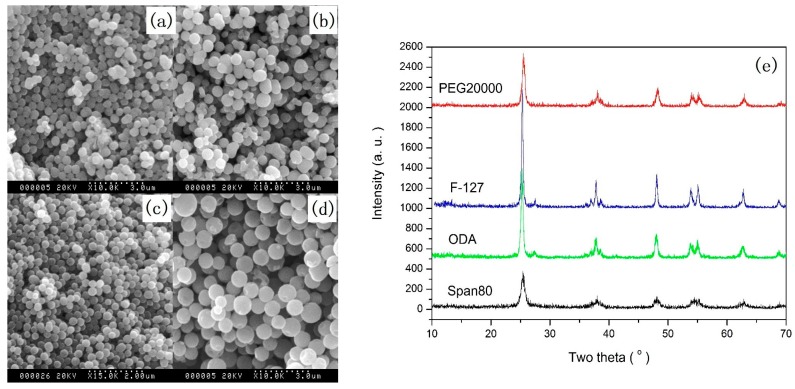
SEM (Scanning electron microscopy) images ((**a**) PEG (polyethylene glycol) 20000, (**b**) ODA (octadecylamine), (**c**) F-127, (**d**) Span80) and the XRD (X-ray diffraction) patterns (**e**) of TiO_2_ spheres synthesized by adding different polymers [37].

Spherical particles with a specific size can be used in many fields, such as photonic crystals, pigments, and so on [15]. In order to obtain monodisperse spherical TiO_2_ particles, many methods have been developed. However, it is still a challenge to control the morphology and size of TiO_2_ microspheres because of the high reactivity of precursors. Increasing the charge of the particle surface and the steric repulsion of the particles are effective methods of controlling the stability of TiO_2_ microspheres [53]. We have reported a simple and reproducible sol-gel method for synthesizing well-defined spherical TiO_2_ particles with diameters within 200–800 nm. In this method, polymers including polyethylene glycol (PEG), poly(ethylene oxide)_106_-poly(propylene oxide)_70_-poly(ethylene oxide)_106_ (F127) copolymer, octadecylamine (ODA), and surfactant Span-80 were used to control the size of TiO_2_ particles [37]. For example, quasi-monodisperse TiO_2_ submicron spheres were synthesized by controlling the hydrolysis of tetrabutyl titanate in ethanol containing the above polymers and small amounts of deionized water. During this process, depending on the used polymer, the transmission time from the transparent solution into white suspension changed from several seconds to minutes. As soon as the transparent solution changed into white suspension, the stirring had to be stopped and the suspension was further aged for 8 h to form quasi-monodisperse TiO_2_ submicron spheres. After high temperature annealing, the spheres were crystallized into the anatase phase. Figure 1 shows the quasi-monodisperse TiO_2_ submicron spheres with different diameters within 200–800 nm, synthesized with different polymers.

#### 2.1.2. Hollow Spheres

Due to high specific surface area and low density, hollow structured materials have been widely used in many fields [54]. TiO_2_ hollow spheres with a well-defined crystal phase are highly desirable for photocatalysis use [55,56,57]. Hollow structured TiO_2_ can be feasibly synthesized by hard template and soft template methods. Compared to the soft template method, the hard template method is simpler, and so it is frequently used.

**Figure 2:**
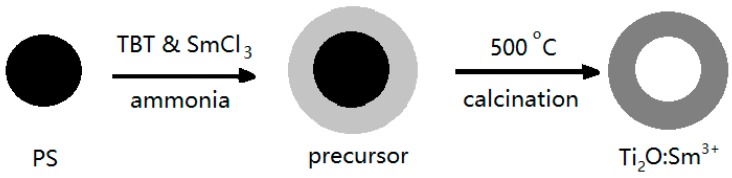
Schematic illustration of the formation mechanism for hollow TiO_2_:Sm^3+^ spheres [29].

**Figure 3:**
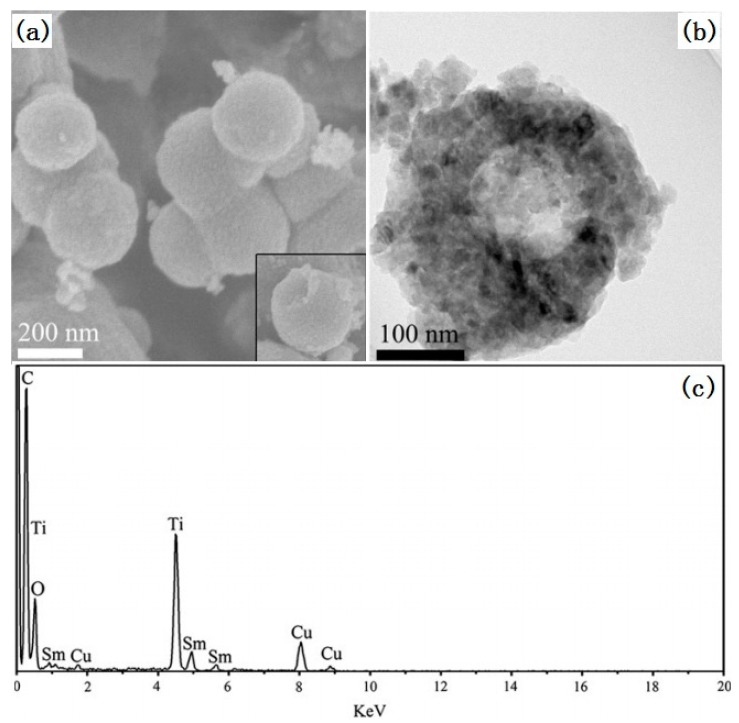
(**a**) SEM image, (**b**) TEM (Transmission Electron Microscopy) image and (**c**) EDS (Energy Dispersive Spectrum) of TiO_2_:Sm^3+^ hollow spheres [29].

An et al. have used polystyrene (PS) spheres as a hard template to prepare hollow Sm^3+^-doped TiO_2_ spheres [29]. The schematic illustration of the formation mechanism is shown in Figure 2. Since the surface of PS spheres obtained by surfactant-free microemulsion polymerization is negatively charged, no additional surface modification of PS spheres is needed for the next coating of TiO_2_. In an ethanol/acetonitrile mixed solvent, a small amount of ammonia was used to induce the hydrolysis of tetrobutyl titanate to form the amorphous Sm^3+^-doped TiO_2_ coating layer on the surface of the PS spheres. After washing with ethanol, drying, and annealing, hollow TiO_2_:Sm^3+^ spheres, as shown in Figure 3, could be obtained.

#### 2.1.3. Porous Spheres

Due to their high surface area, porous materials are very popular for different applications including energy storage, solar cells and catalyzers [58,59,60]. Mesoporous TiO_2_-based materials have attracted much attention for their enhanced reactivity and light harvesting [60]. The macrochannels in mesoporous TiO_2_ particles have served as a light-transfer path that can introduce incident photon flux to the interior surface of the TiO_2_ particles [58]. A mesoporous structure gives light waves more chances to penetrate deep inside the photocatalyst and more light waves are captured. The crystallinity, pore size and composition are important for tuning the properties of mesoporous TiO_2_ spheres [1,8,60]. There has been intensive research concentrated on the design and preparation of porous TiO_2_ materials with unique porosities and tunable pore sizes [61,62,63,64,65].

**Figure 4:**
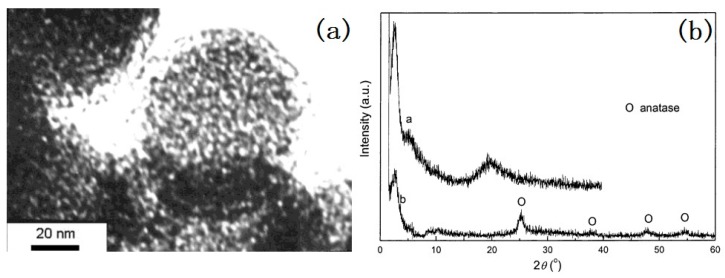
TEM photograph of mesoporous-doped TiO_2_ and XRD patterns (**a**) before hydrothermal and acid treatment, (**b**) after hydrothermal and acid treatment of mesoporous-doped TiO_2_ [64].

We have synthesized mesoporous Ce-doped TiO_2_ spheres by a low-temperature hydrothermal method by using neutral dodecylamine (DDA) as a surfactant and tetrabutyl titanate as a Ti source [64]. To control the rate of hydrolysis of tetrabutyl titanate, a solvent mixture of ethanol and propanol (2:1, *v*/*v*) was used. No additional water was used to initialize the hydrolysis and condensation reaction of the tetrabutyl titanate, due to the used CeCl_3_·7H_2_O containing structured water. After the CeCl_3_·7H_2_O was dissolved, the structured water was released. DDA was able to make the solution alkaline and this made it easy to increase the rate of hydrolysis of the tetrabutyl titanate. However, the dissolution of CeCl_3_·7H_2_O also could result in a decrease of the pH value of solution. Thus, CeCl_3_·7H_2_O could service as not only as a dopant but also as an initiator and buffer. After reaction, a precipitate was formed and it was further refluxed for 2 h at 80 °C in an acid solution to get rid of the template and obtain sphere-like mesoporous Ce-doped TiO_2_ particles with a diameter of 100–1000 nm as shown in Figure 4. The XRD (X-ray diffraction) patterns in Figure 4 show that the TiO_2_ particles are semi-crystalline. The formed anatase crystalline size is very small, about 2–3 nm. In addition, from the TEM (Transmission Electron Microscopy) image, it could be found that the pore structure was worm-like, with a size of 2–3 nm. The Brunauer-Emmett-Teller (BET) surface area of the mesoporous Ce-doped TiO_2_ was 118 m^2^/g, which is much higher than the 9.6 m^2^/g of single-doped TiO_2_ obtained without a surfactant.

**Figure 5:**
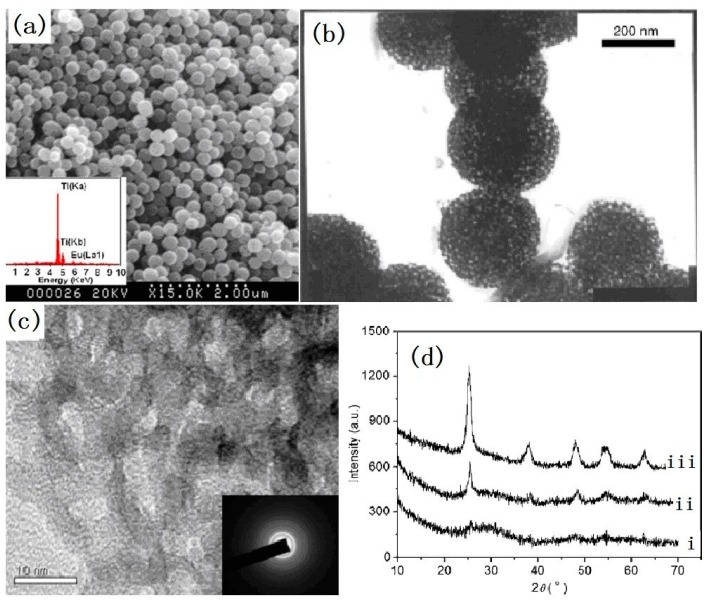
(**a**) SEM image and EDS spectra, (**b**) TEM image, (**c**) high resolution TEM image and corresponding electron diffraction pattern of monodisperse mesoporous after 400 °C calcinations, (**d**) XRD patterns: (i) before calcinations, (ii) after 400 °C calcinations, and (iii) after 500 °C calcinations [28].

The mesoporous Eu-doped TiO_2_ spheres have also been developed by the hydrolysis of tetrabutyl titanate [28]. To increase the thickness of the pore wall, nonionic copolymer Pluronic F-127 was used as a template agent. Europium ethoxide was specially prepared as a doping agent in order to increase the rate of hydrolysis and condensation of the tetrabutyl titanate. Small amounts of water were added to initiate the reaction under stirring. As soon as the solution became slightly white after several minutes, the stirring was stopped. Meanwhile, in order to control the water content, the preparation was conducted under the protection of flowing N_2_. After aging for 24–48 h at 35–40 °C, the suspended particles were filtered and washed with ethanol several times. The final products were obtained after calcination at 400 °C for 4 h. The synthesized Eu-doped TiO_2_ particles have a spherical morphology and a mesoporous structure, with a pore size of 7–10 nm. The special surface area of the phosphor particles is 158 m^2^/g. The high resolution TEM images in Figure 5c show that the pore wall is semi-crystalline that many anatase nanocrystallites are dispersed in the amorphous TiO_2_. The XRD patterns showed in Figure 5d have indicated that no peaks corresponding to the europium compound was detected and no shift of the anatase peaks was observed after doping with Eu^3+^. It can be concluded that Eu^3+^ ions are mainly dispersed in the amorphous TiO_2_ region.

#### 2.1.4. Hollow and Porous Spheres

**Figure 6:**
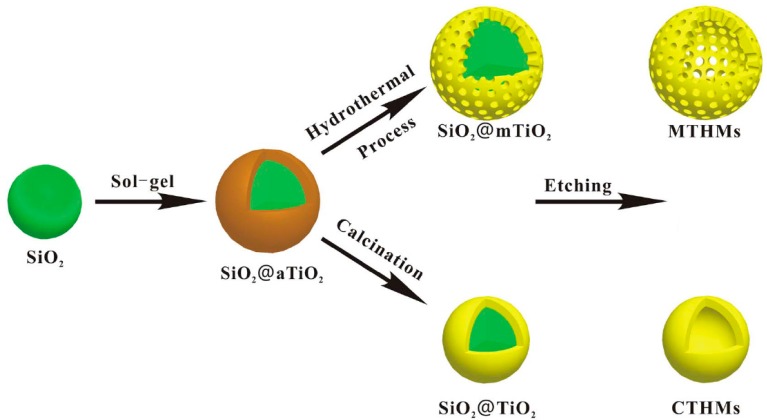
Schematic illustration of the process for the fabrication of the mesoporous TiO_2_ hollow microspheres [39].

**Figure 7:**
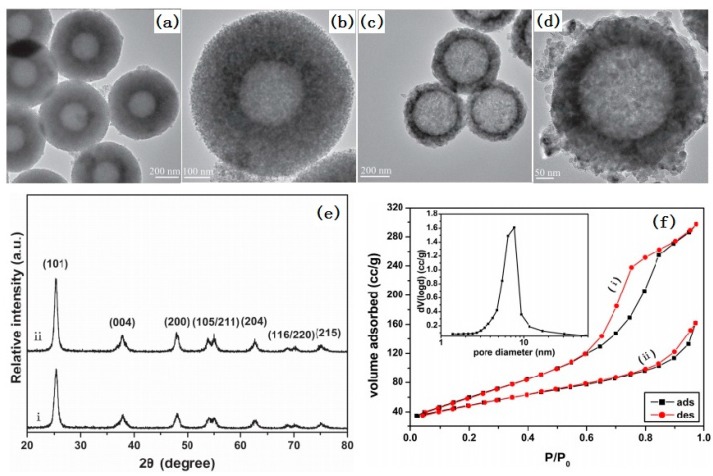
TEM images (**a**,**b**), XRD patterns (**e**-i) and nitrogen adsorption-desorption isotherms (**f**-i) of the sample prepared by the hydrothermal process; TEM images (**c**,**d**), XRD patterns (**e**-ii) and nitrogen adsorption-desorption isotherms (**f**-ii) of the sample prepared by the calcination process [39].

Compared to single hollow spheres, hollow TiO_2_ spheres with a porous shell are more interesting in photocatalysis because they can provide a higher surface area and active site points, decreased diffusion resistance, and increased accessibility to reactants [66]. Several methods have been reported to synthesize hollow TiO_2_ spheres with a porous shell [66,67]. Among these methods, the template method is the most popular. Different sacrificial templates can be used for controlling the size and morphology of such a hollow nanostructure. The template method followed by a hydrothermal or calcination treatment is often used to synthesize hollow TiO_2_ spheres with a crystalline shell [39]. Figure 6 shows a typical process of preparing hollow TiO_2_ spheres with a crystalline shell. In this process, amorphous TiO_2_ was firstly coated onto the surface of SiO_2_ spheres by the sol-gel method in an alkaline solution. Then, the composite microspheres were subjected to a hydrothermal or calcination treatment. Meanwhile, the amorphous TiO_2_ was crystallized into nanocrystals and the mesoporous structure was formed by nanocrystal stacking. Finally, the SiO_2_ core was removed by etching in an alkaline solution. As shown in Figure 7, the sample prepared by hydrothermal treatment had a mean diameter of 620 nm with a 180 nm thick mesoporous TiO_2_ shell. The BET surface area was 231.1 m^2^/g and the pore size was 6.5 nm. However, the sample prepared by calcination had a mean diameter of 440 nm with a 90 nm thick mesoporous TiO_2_ shell. The BET surface area was 158.3 m^2^/g.

### 2.2. 3D Urchin-Like Hierarchical Particles

Urchin-like microspheres possess an epitaxial multilevel structure. The unique micro/nano hierarchical structure has two obvious advantages over single nano-scale or micro-scale structures when they are used as photocatalysts [54,68]. One is that urchin-like TiO_2_ is more efficient at absorbing incidental light because of the increase of multiple-reflection of the hierarchical microspheres [54]. The other is that urchin-like hierarchical TiO_2_ is easy to separate from waste water by the filtration or sedimentation methods. The template-assisted method is a familiar approach to prepare the hierarchical materials. However, it is troublesome to remove templates from products. Impurities are easily introduced into products in the process of utilization and removal of templates. The template-free method is accepted as an ideal strategy which can overcome the drawbacks. Recently, TiO_2_ particles with different hierarchical structures have been successfully fabricated via the template-free method [69,70,71,72,73].

#### 2.2.1. Urchin-Like Hierarchical TiO_2_ Particles

**Figure 8:**
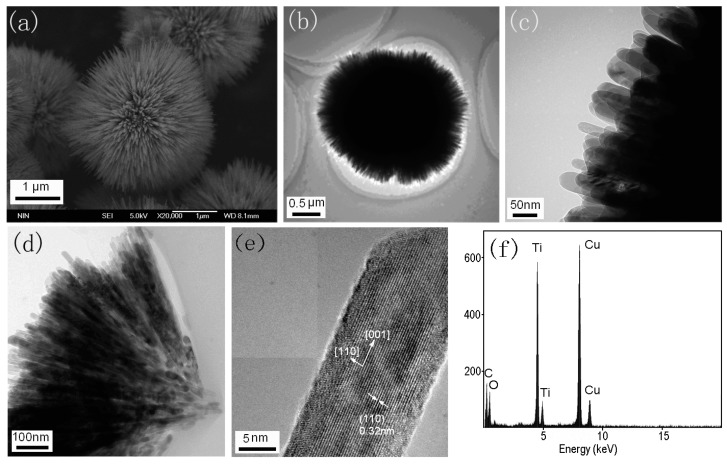
SEM image (**a**), TEM images (**b**–**e**), and EDS (**f**) of the urchin-like hierarchical TiO_2_ [30].

**Figure 9:**
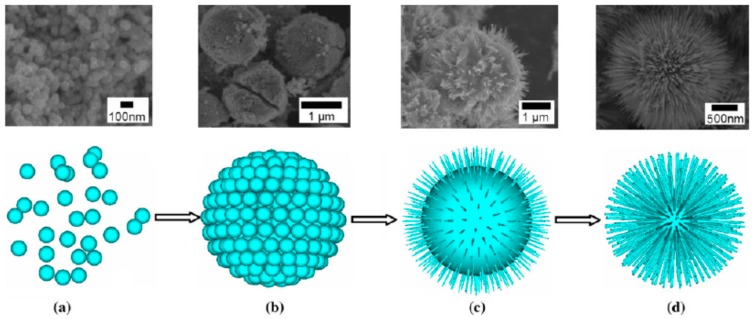
Schematic illustration of the formation process of 3D urchin-like hierarchical TiO_2_: (**a**) nanoparticle; (**b**) microsphere; (**c**) similar urchin-like sphere; (**d**) urchin-like sphere [30].

We have developed a synthesis of a kind of 3D urchin-like TiO_2_ microspheres via a solvothermal method without adding any surfactant or template [30]. Tetrabutyl titanate and titanium tetrachloride (TiCl_4_) aqueous solution were used as the reactant, and toluene was used as the solvent. The solvothermal reaction took place in a Teflon-lined autoclave at 150 °C for 24 h. Sea-urchin-like 3D hierarchical TiO_2_ microspheres with a uniform size of 2.5–3.0 μm were obtained (Figure 8). The 3D hierarchical microspheres were made of single crystalline rutile nanoneedles with diameters about 20–40 nm, which grew radially from the core of the microspheres. The morphology and crystal phase of the 3D hierarchical TiO_2_ microspheres could be influenced by some factors, such as the ratio of tetrabutyl titanate to TiCl_4_, the solvothermal temperature, and so on. By tracing the particle morphology change by SEM and XRD techniques, we concluded that the formation of 3D hierarchical TiO_2_ microspheres mainly concerned three steps, i.e., nucleation, self-assembly, dissolution and recrystallization, as depicted in Figure 9. In the nucleation stage, nanoparticles were formed. Then, the nanoparticles assembled into microspheres. Finally, the microspheres gradually changed into the urchin-like hierarchical structure by dissolution and recrystallization.

#### 2.2.2. Cr Doped Urchin-Like Hierarchical TiO_2_ Particles

**Figure 10:**
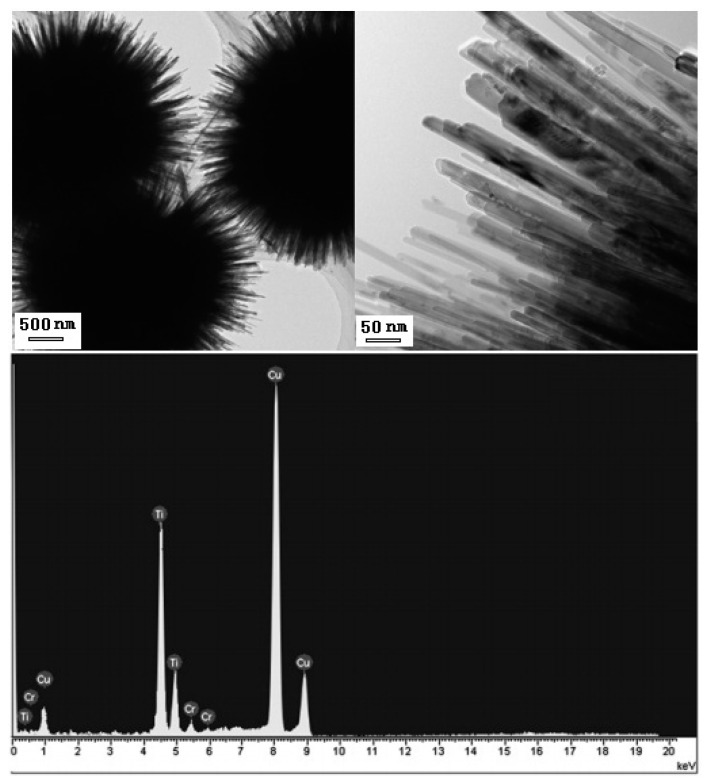
TEM images and EDS spectra of Cr-doped urchin-like TiO2 particles [73].

Urchin-like Cr-doped TiO_2_ particles could be also synthesized by the same solvothermal method described above in a solution of titanium tetrabutyl titanate dissolving CrCl_3_ [73]. The morphology of Cr-doped TiO_2_ particles is characterized by SEM images shown in Figure 10. The mean particle size of the hierarchical microspheres can be adjusted within 1–5 μm and the diameter of the nanorods is about 20–30 nm. The EDS results showed that the content of the Cr element in the Cr-doped TiO_2_ particles was ~2.9 mol %.

#### 2.2.3. Noble Metal Nanoparticle-Decorated 3D Urchin-Like TiO_2_ Particles

**Figure 11:**
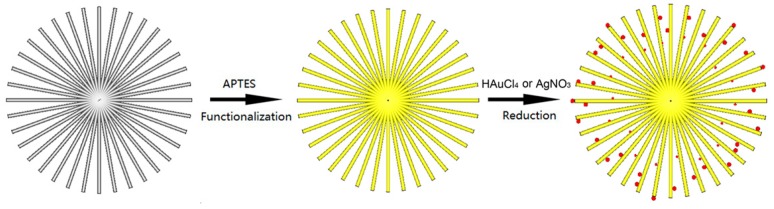
Schematic illustration of the synthesis process of urchin-like TiO_2_ decorated with Au or Ag nanoparticles [31].

**Figure 12:**
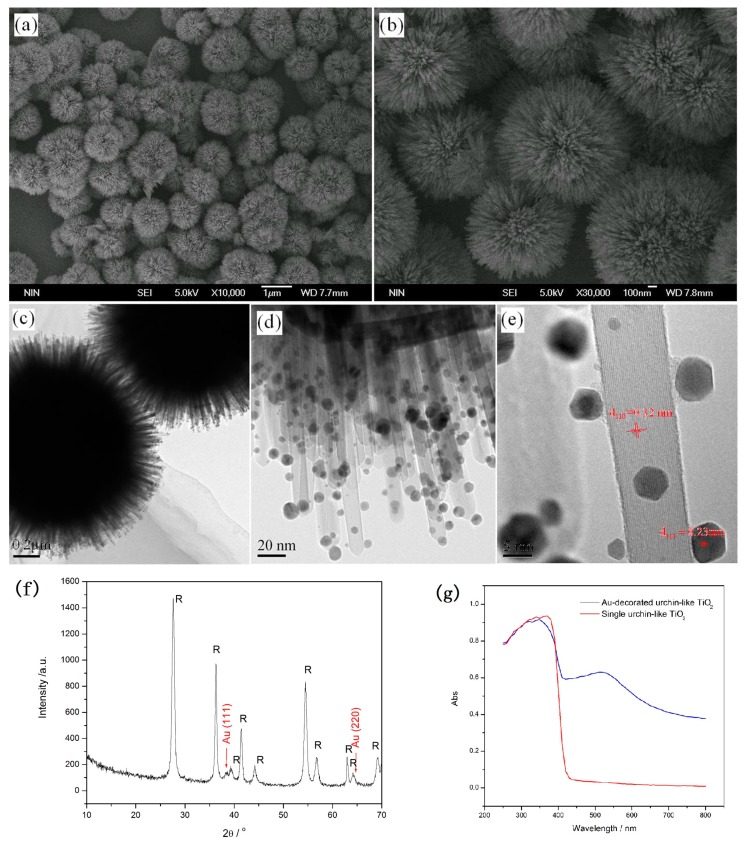
SEM images (**a**,**b**), TEM images (**c**–**e**), XRD pattern (**f**) and UV-Vis absorption spectra (**g**) of Au-decorated 3D urchin-like TiO_2_ nanostructures [31].

Noble metal nanoparticle-decorated semiconductors are interesting for photocatalysis because of their combined properties [74]. Decorating the noble metal nanoparticles (e.g., Au, Ag and Pt) onto the surface of TiO_2_ is an effective method to improve the photocatalytic activity because not only light-harvesting efficiency can be enhanced due to the surface plasmon resonance of noble metal nanoparticles, but the recombination of surface radicals can also be slowed down by capturing photogenerated electrons of noble metal nanoparticles [75,76,77,78,79]. Figure 11 shows a schematic illustration of 3D urchin-like hierarchical TiO_2_ microspheres decorated with Au nanoparticles via a two-step wet-chemical process [31]. In the first step, the surface of the urchin-like TiO_2_ microspheres was modified with APTES (3-aminopropyl-triethoxysilane) that possess amidocyanogen. Then, the modified particles were decorated with Au nanoparticles in HAuCl_4_ aqueous solution by the reduction of NaBH_4_. Since the amidocyanogen could interact with Au nanoparticles by a weak covalent bond, Au nanoparticles were closely attached to the surface of the TiO_2_ nanostructures, as shown in Figure 12. It was seen that the Au nanoparticles with diameters of about 2–10 nm mainly adhered to the surface of the needles uniformly. Most of the Au nanoparticles possess a rhombic dodecahedra structure. The UV-Vis (ultraviolet-visible) spectra show an absorption band located at the wavelength of about 530 nm due to the surface plasmon resonance of Au nanoparticles.

**Figure 13:**
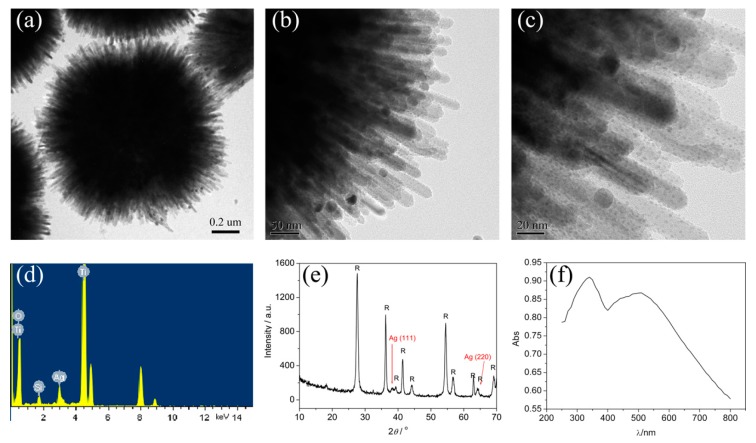
TEM images (**a**–**c**), EDS (**d**), XRD (**e**), and UV-Vis absorption spectrum (**f**) of Ag-decorated 3D urchin-like TiO_2_ nanostructures [31].

The urchin-like TiO_2_ microspheres decorated with Ag nanoparticles could be also prepared by the similar process. As shown in Figure 13, the Ag nanoparticles with diameters of about 2 nm are decorated on the TiO_2_ nanoneedles homogeneously. A broad absorption band at around 500 nm, corresponding to the surface plasmon resonance of the Ag nanoparticles, appears in the UV-Vis absorption spectrum (Figure 13f).

#### 2.2.4. Core/Shell-Structured Bimetallic Nanoparticle-Decorated 3D Urchin-Like Hierarchical TiO_2_ Particles

**Figure 14:**
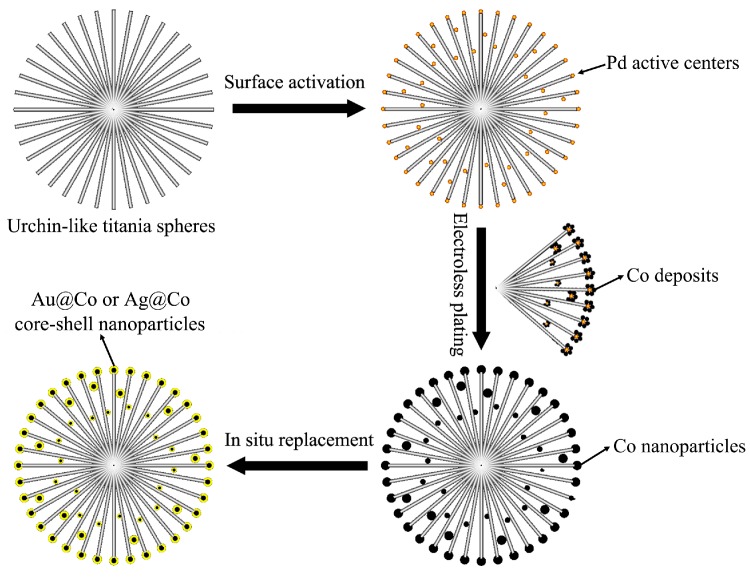
A schematic synthesis process of urchin-like TiO_2_ decorated with core/shell-structured Co@Au or Co@Ag bimetallic nanoparticles [32].

Bimetallic nanostructures often show a more excellent comprehensive performance over their monometallic counterpart [80]. Especially core/shell bimetallic nanostructures with a magnetic core and a noble-metallic shell have aroused researchers’ interest [81,82,83,84,85]. The magnetic core can offer a drive force for the recycling of samples, while the noble-metal shell can improve the optical properties [85]. Figure 14 shows a typical preparation process of a kind of 3D urchin-like hierarchical TiO_2_ decorated with a bimetallic core/shell nanoparticle (Co@Au and Co@Ag). The preparation includes three steps, i.e., surface activation, electroless plating and in-situ replacement [32]. First, the surface of the urchin-like TiO_2_ microspheres was activated by implanting Pd nanodots. Then, Co nanoparticles were formed and adhered to the nanoneedle surface of the urchin-like TiO_2_ microspheres by electroless plating. Finally, Ag or Au were further formed and coated on the surface of the Co nanoparticles by an in-situ replacement process.

**Figure 15:**
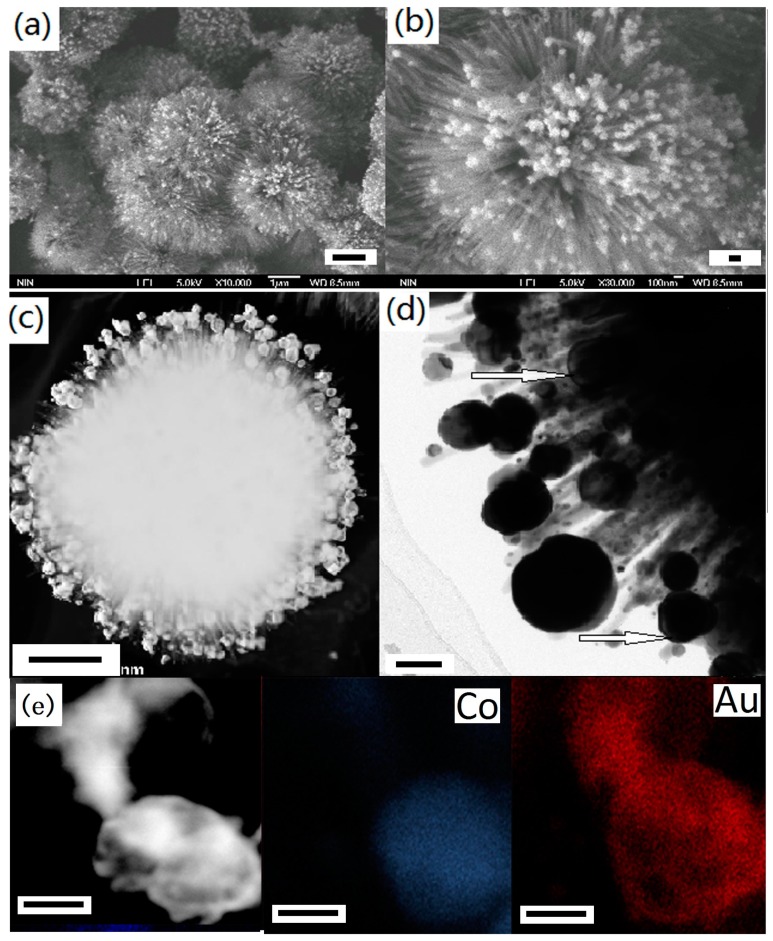
The SEM and TEM images of Co@Au/TiO_2_ composites: (**a**,**b**) SEM images, (**c**,**d**) TEM images, (**e**) high-resolution TEM images; (**e**) the local elemental mapping of Co and Au (Scale bar = 1 μm for (**a**); scale bar = 100 nm for (**b**); scale bar = 500 nm for (**c**); scale bar = 100 nm for (**d**); scale bar = 50 nm for (**e**)) [32].

The SEM and TEM images in Figure 15 show the morphology of Co@Au/TiO_2_ composites. It can be seen that many core/shell nanostructured nanoparticles with diameters of 10–80 nm are attached to the surface of TiO_2_ nanorods. The images of the elemental mapping of core/shell nanoparticles further identify that the core is Co and the shell is Au. The size and distribution of the bimetallic particles can be adjusted by controlling the ratio of Co to TiO_2_ during the electroless plating process. The thickness of the Au or Ag shell could be controlled by adjusting the concentration of HAuCl_4_ or AgNO_3_ in the solution and the reaction time. Both the Co@Au/TiO_2_ and Co@Ag/TiO_2_ particles showed good response to an applied external magnetic field [32].

#### 2.2.5. Photocatalytic Activity of Urchin-Like Hierarchical TiO_2_ and Their Composites

Although TiO_2_ is an ideal candidate for photocatalysis because of its strong oxidization, harmlessness to surroundings, chemical inactivity, good stability and low cost, the main weakness of TiO_2_ is the lack of visible light response due to the large band gap. Therefore, the question of how to increase the efficiency of visible light harvesting is an important research topic in this field [1]. Although single controlling the morphology of TiO_2_ materials cannot increase the efficiency of visible light harvesting, it is possible to enhance the visible light harvesting of TiO_2_ composites by combining ion doping or noble metal decoration with morphology control. Ion doping or noble metal decoration can induce or increase the visible light absorption of TiO_2_, while the absorption effect can be further enhanced by other effects from material morphology, such as decreased light scattering or increased multiple reflection, etc.

**Figure 16:**
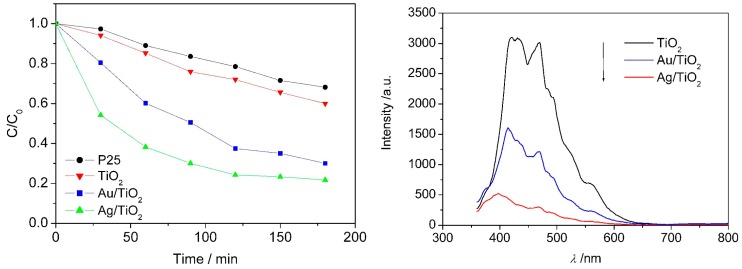
Photodegradation curves of MB (methyl blue) in the presence of P25, TiO_2_, Au/TiO_2_ and Ag/TiO_2_ (**left**); Photoluminence spectra (*λ*_ex_ = 215 nm) of pure urchin-like TiO_2_, Au/TiO_2_ and Ag/TiO_2_ (**right**) [31].

**Figure 17:**
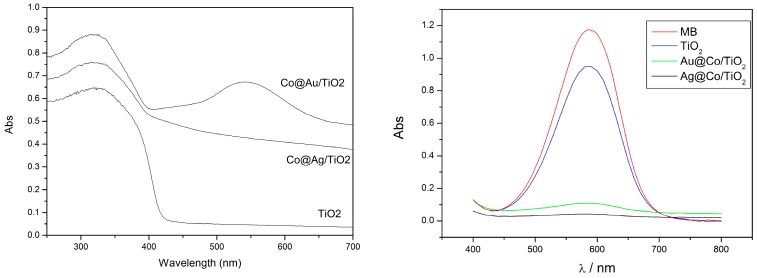
UV-Vis absorption spectra of TiO_2_, Co@Au/TiO_2_ and Co@Ag/TiO_2_ (**left**); UV-Vis absorption spectra of MB before and after degradation with TiO_2_, Co@Au/TiO_2_ and Co@Ag/TiO_2_ for 15 min at room temperature (**right**) [32].

The photocatalytic efficiency of commercial P25, urchin-like TiO_2_ and Au or Ag-decorated urchin-like TiO_2_ was evaluated by degrading MB (methyl blue) under UV-Vis light irradiation. It was found that the photocatalytic degradation efficiency under the same conditions followed the order: Ag/TiO_2_ > Au/TiO_2_ > TiO_2_ > P25 (Figure 16). By the photoluminescence spectra, Au or Ag nanoparticles decorated on the surface of TiO_2_ were demonstrated to be able to effectively capture photogenerated electrons and prevent electron/hole recombining (Figure 16). In addition, the urchin-like micro/nano hierarchical structure also may increase the visible light harvesting efficiency by the multiple-reflection of nanoneedles. These should be responsible for the enhanced photocatalytic efficiency of urchin-like TiO_2_ microspheres after decoration with Au or Ag nanoparticles. Similarly, the light-harvesting efficiency could also be enhanced by decorating with Co@Au or Co@Ag bimetallic nanoparticles, as shown in Figure 17. As a result, the photocatalytical efficiency of urchin-like TiO_2_ was enhanced obviously, as shown in Figure 17, by the experiment of decolorizing methyl blue (MB) solution.

## 3. Summary

Based on our group’s research work, we provided a brief review of the synthesis of TiO_2_ with different morphologies and the photocatalytic properties of urchin-like TiO_2_, noble metal nanoparticle-decorated 3D urchin-like TiO_2_ and core/shell-structured bimetallic nanoparticle-decorated 3D urchin-like hierarchical TiO_2_. The examples of the fabrication of solid spheres, hollow spheres, porous spheres, and porous and hollow microspheres of anatase TiO_2_-based materials were introduced. The synthesis and photocatalytic efficiency of urchin-like rutile TiO_2_, urchin-like rutile TiO_2_ nanostructures decorated with Au or Ag nanoparticles and core/shell-structured bimetallic nanoparticles (Co@Au and Co@Ag) were especially introduced. The results of photocatalytic tests show that 3D urchin-like hierarchical structures have unique merits in the efficient harvesting of solar light, and decorating Au, Ag or bimetallic nanoparticles on the surface of 3D urchin-like TiO_2_ can promote photoinduced charge-carrier separation.
